# Hippocampal Hyperexcitability is Modulated by Microtubule-Active Agent: Evidence from *In Vivo* and *In Vitro* Epilepsy Models in the Rat

**DOI:** 10.3389/fncel.2016.00029

**Published:** 2016-02-09

**Authors:** Fabio Carletti, Pierangelo Sardo, Giuditta Gambino, Xin-An Liu, Giuseppe Ferraro, Valerio Rizzo

**Affiliations:** ^1^Department of “Experimental Biomedicine and Clinical Neuroscience” (Bio.Ne.C.), “Sezione di Fisiologia Umana G. Pagano”, University of PalermoPalermo, Italy; ^2^Post-graduate School of Nutrition and Food Science, University of PalermoPalermo, Italy; ^3^Department of Neuroscience, The Scripps Research InstituteJupiter, FL, USA

**Keywords:** hippocampus, epilepsy, maximal dentate activation, microtubule, electrophysiology, nocodazole, paclitaxel

## Abstract

The involvement of microtubule dynamics on bioelectric activity of neurons and neurotransmission represents a fascinating target of research in the context of neural excitability. It has been reported that alteration of microtubule cytoskeleton can lead to profound modifications of neural functioning, with a putative impact on hyperexcitability phenomena. Altogether, in the present study we pointed at exploring the outcomes of modulating the degree of microtubule polymerization in two electrophysiological models of epileptiform activity in the rat hippocampus. To this aim, we used *in vivo* maximal dentate activation (MDA) and *in vitro* hippocampal epileptiform bursting activity (HEBA) paradigms to assess the effects of nocodazole (NOC) and paclitaxel (PAC), that respectively destabilize and stabilize microtubule structures. In particular, in the MDA paroxysmal discharge is electrically induced, whereas the HEBA is obtained by altering extracellular ionic concentrations. Our results provided evidence that NOC 10 μM was able to reduce the severity of MDA seizures, without inducing neurotoxicity as verified by the immunohistochemical assay. In some cases, paroxysmal discharge was completely blocked during the maximal effect of the drug. These data were also in agreement with the outcomes of *in vitro* HEBA, since NOC markedly decreased burst activity that was even silenced occasionally. In contrast, PAC at 10 μM did not exert a clear action in both paradigms. The present study, targeting cellular mechanisms not much considered so far, suggests the possibility that microtubule-active drugs could modulate brain hyperexcitability. This contributes to the hypothesis that cytoskeleton function may affect synaptic processes, relapsing on bioelectric aspects of epileptic activity.

## Introduction

Epilepsy is a neurological disorder characterized by recurrent seizure attacks due to synchronous neuronal firing or increased neuroexcitability of specific brain regions (Hauser and Hesdorffer, 1990; Stafstrom and Carmant, 2015). The broad spectra of epilepsy-related neurobiological targets include, overall, synaptic components, neurotransmitter mechanisms or metabolic pathways (Bialer et al., 1996, 2001; Scharfman, 2007; Roseti et al., 2013; Moccia et al., 2015), but only few studies have investigated the potential role of the cytoskeleton. In this regard, hippocampal epilepsy is often associated with sprouting of principal cell axons in both humans and animal models (Bausch, 2005). On this point, microtubule cytoskeletal alterations underlying aberrant axonal sprouting have been implicated in the development of posttraumatic epilepsy (Larner, 1995; McKinney et al., 1997; Chuckowree and Vickers, 2003; Wilson et al., 2012). The neuronal microtubule cytoskeleton underpins many cellular processes including protein transport, cell division, neurotrophic support but it also plays a fundamental role in regulating voltage-gated ion channels activity and the affinity of several neurotransmitters for their receptors (Whatley and Harris, 1996). Noteworthy, disruptive mutations in α- or β-tubulin may lead to neurological diseases in both the central and peripheral nervous system and dysfunctions of the microtubule cytoskeleton likely determine aberrant neurotransmission (Craddock et al., 2010; Gardiner and Marc, 2010; Millecamps and Julien, 2013). Furthermore, microtubules have been found to be extremely important for the general bioelectric activity of neurons serving as biological electrical wires that can transmit and amplify electric signals via the flow of condensed ion clouds (Craddock et al., 2010). Therefore, even if the role of microtubule dynamics on neuroexcitability and synaptic communication has been already suggested (Whatley and Harris, 1996; Gardiner and Marc, 2010), it is still to be elucidated whether this may be directly related to hyperexcitability and hence, importantly contribute to epileptic phenomena. In order to achieve a deeper insight on the impact of microtubules on neuronal hyperexcitability in mammalian brain, we studied the effect of the pharmacological manipulation of the microtubule polymerization on two different electrophysiological acute models of hippocampal epilepsy in the rat. To this purpose, we administered nocodazole (NOC) and paclitaxel (PAC), previously chosen to study hippocampal processes for their reported action on cytoskeleton (Craddock et al., 2010; Fanara et al., 2010), but also recognized in the clinical practice as anti-mitotics in different forms of cancer (Jordan and Wilson, 2004). In detail, NOC is mainly known for its affinity to the major microtubule structural protein tubulin, reducing microtubule instability and promoting depolymerization. Conversely, PAC binds to tubulin at a different site and hyperstabilizes polymerized microtubules (Gotaskie and Andreassi, 1994; Dinter and Berger, 1998; Marx et al., 2007). We exploited two drug-free models of epileptiform activity, the *in vivo* electrically-induced maximal dentate activation (MDA; Stringer and Lothman, 1992; Carletti et al., 2015), and the hippocampal epileptiform bursting activity (HEBA), characterized by *in vitro* neuronal burst firing evoked by changes in the electrolytic concentrations of cerebrospinal fluid (Sokolova et al., 1998; Sardo et al., 2012). Noticeably, the MDA study was primarily conducted with experimenters blind to the drugs identity to prevent the possibility of bias from interpretation of electrophysiological outcomes. Then, *in vitro* assessment followed for a deeper understanding of what observed *in vivo*. Coupling *in vivo* and *in vitro* methods allowed us to evaluate the effects of perturbing the microtubule assembly process on the genesis and maintenance of epileptic activity, in the hippocampus, in both intact neuronal network and isolated brain slices. On the whole, this study can be placed in the research field of synaptic mechanisms underlying pathophysiological alterations of neural transmission, conceivably relapsing on hyperexcitability phenomena.

## Materials and Methods

### *In Vivo* MDA Model

#### Animals

Male Wistar rats, weighing 240–280 g, were anesthetized with urethane (1.2–1.4 g/kg intraperitoneally, i.p.; Maggi and Meli, 1986; Hara and Harris, 2002). The animals were positioned in a stereotaxic apparatus for skull exposition (David Kopf Instruments, Tujunga, CA, USA). The body temperature was maintained at 37–38°C using a heating pad; hearth rate and pupil diameter were monitored during all the experimental session. A craniotomy was performed to expose a wide area of the right cerebral cortex; then, the dura was reflected. A stimulating depth electrode (coaxial bipolar stainless steel electrode: external diameter 0.5 mm; exposed tip 25–50 μm) was placed in the angular bundle (AB) on the right side according to the stereotaxic coordinates of the Atlas of Paxinos and Watson (1998; AB: 1 mm anterior to the interaural line; 3–5 mm dorsal to it and 4.4 mm lateral to the midline). A glass recording electrode, filled with 1% fast Green in 2 M NaCl, was stereotaxically placed in the dentate gyrus (DG) on the right side (DG: 6 mm anterior to the interaural line; 1.8 mm lateral to the midline and 3.0 mm ventral to the cortical surface). The site for intracerebroventricular (ICV) injection was localized into the right cerebral ventricle (RV; 0.8 mm posterior to the bregma; 1.4 mm lateral to the midline and 3.3 mm from the dura). The animal was grounded through a subcutaneous Ag/AgCl wire in the scapular region. The bioelectric activity of the structure examined was amplified and recorded through a low level DC pre-amplifier (Grass 7B, West Warwick, RI, USA), then processed using the SciWorks package, version 5.0 (Datawave Technologies, Longmont, CO, USA). All procedures were performed in strict accordance with the European directive 2010/63/EU and the institutional guidelines, authorized by the Italian Ministry of Health (authorization n° 258-95-A) and approved by the Committee for the Protection and Use of Animals of the University of Palermo. All efforts were made to minimize animal suffering and to reduce the number of animals used.

#### Maximal Dentate Gyrus Activation and Ictal Events Identification

**Figure 1:**
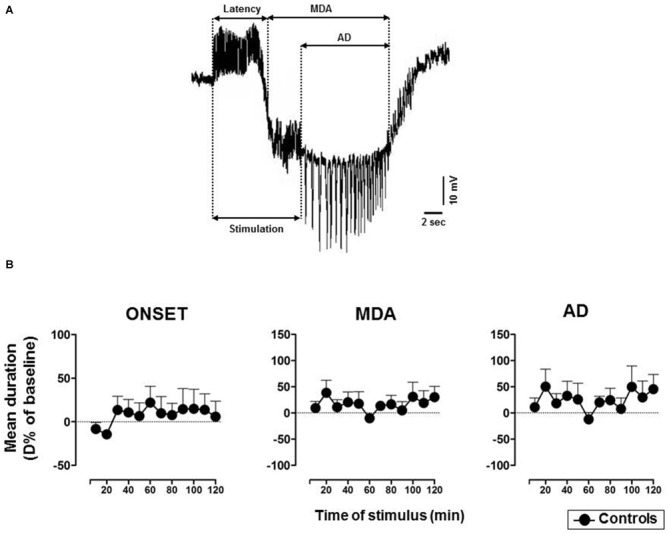
**(A)** Representative maximal dentate activation (MDA) trace. Measurements of time of onset (latency), duration of maximal dentate gyrus (DG) activation (MDA) and after discharge (AD) during and after a stimulus train (400 μA, 20 Hz) delivered for 10 s to the angular bundle (AB).** (B)** Time course of MDA parameters in vehicle-treated controls. Each value represents the mean duration of D% of baseline ± SEM.

The technique originally used by Stringer and Lothman (1992) to trigger the activation of the DG was modified as described in our previous research (Sardo et al., 2006, 2008, 2009). In detail, 10 s duration trains of 20 Hz stimuli were given through the AB stimulating electrode. Individual stimuli consisted of 0.3 ms biphasic pulses. The stimulus intensity was initially below that necessary to elicit response and then, increased in 100 μA steps until MDA occurred (threshold intensity). A stimulus train was administered every 2 min until MDA appearance and then every 10 min for up to 3 h. A stimulus intensity 100 μA higher than the threshold intensity for the stimulations was used. In every case, the stimulation intensity varied from 200 μA to 400 μA. MDA was defined by a shift of the extracellular potential in DC-coupled recordings as well as by the presence of bursts of population spikes of 20–40 mV in DC-coupled trace. First, we took into consideration the % of responses to AB stimulation to evaluate the possible drug-induced suppression of paroxysmal events. Furthermore, the following parameters were analyzed to assess changes of epileptic activity: (i) the time of onset (or latency) of the MDA was considered as the time from the beginning of AB stimulation to the midpoint of the shift of the DC potential; (ii) the total duration of the MDA was measured from the midpoint of the shift of the DC potential to the point at which the evoked paroxysmal activity abruptly ceased; (iii) the afterdischarge (AD) duration was measured from the end of AB stimulation to the end of the epileptiform activity (Figure 1A). The time of onset represents an indicator of the susceptibility of the DG to respond to stimulation; MDA and AD quantify the extent of epileptic discharge.

The correct positioning of electrodes was verified through iontophoretic Fast Green injection (50 μA for 10 min) and a small electrolytic lesion (20 mA for 10 s), as previously described (Sardo et al., 2008).

#### Drug Treatment

NOC, PAC and all reagents were purchased from Sigma Chemical Co., (Sigma, St. Louis, MO, USA), if not differently specified. The study was performed by blind experimenters on four groups of rats (*n* = 6 each): in the 1st (controls) the animals were studied for a period of about 3 h in order to verify possible modifications of MDA parameters due to the repetitive stimulations. In the 2nd group the animals received administration of vehicle (1 μL of 15% dimethyl sulfoxide solution, DMSO, ICV). In the 3rd and the 4th group, 1 μL of either NOC or PAC were injected ICV in order to reach a 10 μM final concentration in the cerebrospinal fluid for both drugs. For all the injections, a 2.5 μL Hamilton microsyringe (Supelco, Bellefonte, PA, USA) connected to a microinjection pump (2Biological Instruments, Besozzo, VA, Italy) was used. All injections were delivered at a rate of 0.5 μL/min and the needle was left in place for one additional minute following the infusion to allow the diffusion of the drug away from needle tip. ICV injections were carried out after five stimulations that produced a stable MDA (baseline period) and the following observation last up to 120 min. The dosages of administration were used on the basis of previous studies on these drugs and pilot experiments (Furukawa and Mattson, 1995; Puthanveettil et al., 2008; Miller et al., 2015).

#### Statistical Analysis

The chi-square (χ^2^) test was used to compare the % of responses to AB electrical stimulation following each drug treatment within the same experimental group and also between treated groups and vehicle-treated controls.

For each studied parameter (the time of onset, MDA or AD durations), data from each animal were expressed as % difference (D%) vs. the baseline value measured in the last MDA response preceding vehicle (in the 2nd group) or drug (in 3rd and 4th groups) injection (Figure 1B). Then, in each group D% data were averaged (mean ± SD) on the basis of time elapsed from the first stimulation following the treatment and then plotted as mean D% ± SEM (see Figure legends). For significant changes, maximum mean D% and the related mean absolute values are reported. The time course of response parameters was analyzed using a two-way analysis of variance (ANOVA), since the occurrence of drug-induced suppression of paroxysmal response did not allow the use of a repeated measures ANOVA. This analysis was followed by Bonferroni *post hoc* test. A between-treatments comparison was made to assess the effects of the treatments in the 3rd, and 4th groups respectively vs. vehicle-treated controls. Differences were considered statistically significant when *P* was less than 0.05.

#### Immunohistochemistry

At the end of the MDA protocol (namely 2 h after NOC ICV injection), for histological verification of NOC ICV effects, six animals received an overdose of pentobarbital i.p. and were then whole-body perfused with normal saline followed by 10% buffered formalin. The brains were removed, postfixed in the same fixative overnight and then cryoprotected in 30% sucrose/PBS. Subsequently, the brains were sliced in serial sections, used for the following immunohistochemical assay according to established procedures (Morterá and Herculano-Houzel, 2012; Woeffler-Maucler et al., 2014; Kadakkuzha et al., 2015). Four representative 40 μm floating horizontal sections containing DG, RV and entorhinal cortex (EC; from 3.0 to 5.1 mm, ventral to cortical surface) were rinsed at least three times in PBS, pretreated with 0.5% Triton X-100-PBS for 20 min at room temperature to rupture membrane, and then immersed in 10% normal goat serum (NGS) in PBS for 60 min followed by incubation with primary antibodies containing 3% NGS, and 0.1% Triton X-100 in PBS for 24 h on a rotating shaker at 4°C. After primary incubation, sections were washed four times with 0.1% Triton X-100 in PBS and then incubated in secondary antibodies containing 3% NGS in PBS for 1 h on a rotating shaker at room temperature. Primary antibodies consisted of rabbit anti-NeuN (1:200, ABN78; Millipore), and mouse anti-β-tubulin (1:500, T4026; Sigma-Aldrich). Brain sections from control and treated animals were analyzed per region (*n* = 3 brains per group). Secondary antibodies consisted of anti-rabbit Alexa Fluor 488 (1:1500; Invitrogen) and anti-mouse Alexa Fluor 546 (1:1500; Invitrogen). For counterstaining of nuclei, the sections were stained using Hoechst Stain solution.

#### Confocal Microscopy and Data Analysis

Labeled sections were mounted on slides sequentially according to the rat brain atlas with a fluoro-gel with DAPI (Fluoro-Gel with DAPI; Electron Microscopy Sciences), cover slipped, and imaged using a Zeiss LSM 780 confocal microscope system. For each section, a 10× tile scan image was obtained and followed by image captures with 63× objective of random positions in the regions of interest from both hemispheres. Representative images were chosen from the same positions in vehicle and NOC-treated rats. Only projection images were shown. The NeuN positive neurons as well as DAPI positive neurons were counted using Image J software. The ratio of NeuN positive neuron number/DAPI number in each image (mean ± SEM) was calculated and analyzed by a *t*-test to outline the presence of histological modifications in treated vs. control brains. Four to six 63× images from each region were used for the analysis.

### *In Vitro* HEBA Model

#### Hippocampal Epileptiform Bursting Activity in Rat Brain Slices

Brain slices were obtained from 4- to 6-week-old male Wistar rats. In detail, rats were deeply anesthetized with halothane, then decapitated; brains were carefully removed and glued to the stage of a vibroslicer (Vibroslice–Campden, mod. MA752). Slicing was carried out in ice-cold (3–4°C) oxygenated artificial cerebrospinal fluid (aCSF) containing (in mM): 124 NaCl, 3 KCl, 1.25 NaH_2_PO_4_, 1.8 MgSO_4_, 1.6 CaCl_2_, 26 NaHCO_3_, and 10 Glucose, pH 7.4. aCSF was constantly bubbled with a 95% O_2_, 5% CO_2_ gas mixture. From the resulting horizontal slices (350–400 μm thickness), only the hippocampi were immediately transferred to a holding chamber containing normal oxygenated aCSF and held at 33°C. After 1 h recovery, a slice was rapidly transferred to a standard Interface Recording Chamber, continuously perfused (2 ml/min) with normal aCSF at 35 ± 0.5°C; a warmed, humidified 95% O_2_–5% CO_2_ vapor was maintained over the exposed surface of the slice. In order to induce hippocampal bursting activity, the slice was then perfused with aCSF, containing modified concentrations of Ca^2+^ (1.0 mM), Mg^2+^ (1.5 mM) and K^+^ (8.0 mM; Sokolova et al., 1998). Electrophysiological recordings from the CA1 hippocampal subregion were performed by means of borosilicate glass microelectrodes (5–7 MΩ), filled with 2 M NaCl, and lowered to a depth of 100–150 μm below the cut surface of the slice, at which ionic changes accompanying the epileptiform acivity are maximal (Sokolova et al., 1998). Electrical activity was recorded using a Multiclamp 700B amplifier (Axon Instruments, Molecular Devices, CA, USA, 300 Hz–3 kHz band pass). The raw activity signal was video monitored through a Tektronix 5113 oscilloscope (Beaverton, OR, USA). The resulting Transistor–Transistor Logic pulses were used to trigger a digitizing oscilloscope (Gould 500 DSO) for displaying selected full waveforms of discriminated impulses. The raw electrical activity was also digitally converted; in addition to fully storing it on a computer for off-line analysis, raw activity was passed through a software window discriminator and digital signals were on-line displayed. Furthermore, a ratemeter histogram of burst activity was continuously displayed and updated each 5 s on the computer screen together with a counter window, to detect on-line variations of paroxysmal bursting rate. All computer operations were performed using the pClamp package, version 10.5.0 (Molecular Devices, Berthoud, CO, USA).

#### Drug Treatments

After a steady epileptiform bursting discharge (control period) was obtained and maintained for at least 10 min, the slices were perfused for 10 min with modified aCSF containing NOC 10 μM. At the end of drug administration, slices were again perfused with drug-free modified aCSF for 10 min (wash-out period). The duration and dosages of administration were chosen taking into consideration previous studies on these drugs, the reported equilibration time course of other antiepileptic drugs and pilot experiments in hippocampal slices at the depth of recording (Furukawa and Mattson, 1995; Petrini et al., 2004; Puthanveettil et al., 2008; Sardo et al., 2012).

#### Statistical Analysis

**Figure 2:**
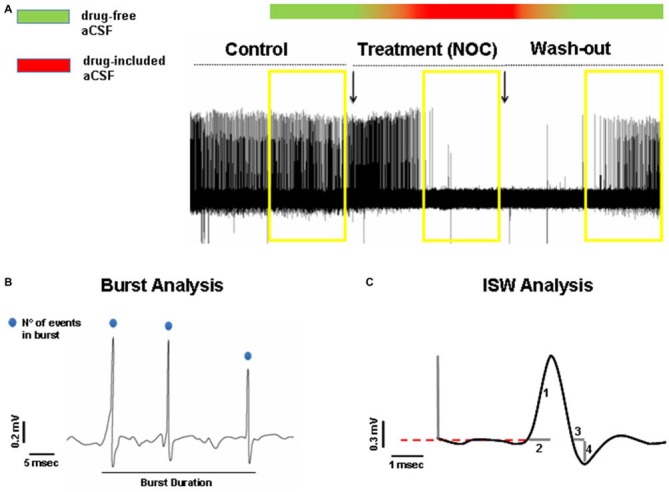
**(A)** Representative hippocampal epileptiform bursting activity (HEBA) trace. The yellow boxes indicate the periods (5 min each) of control, treatment (NOC for nocodazole) and wash-out, taken into consideration for analysis. The bar on the top represents the continuous perfusion with modified aCSF, containing drug-free aCSF (green) or drug-included aCSF (red). Chromatic fading along the bar indicates the progressive filling of the recording chamber with the different aCSF solutions. **(B)** Measurements of burst parameters, i.e., burst duration and the number (N°) of events in burst. **(C)** Measurements of intraburst single spike waveform (ISW) parameters: peak amplitude indicated as (1), time to peak indicated as (2), time to antipeak indicated as (3) and antipeak amplitude indicated as (4).

In order to detect statistically significant drug-related changes, we analyzed extracellular bursting activity of drug treatment periods, then compared vs. control values and wash-out periods, respectively. As control activity we took into account 5 min recorded immediately before switching to drug-included aCSF and as wash-out period, the last 5 min recorded after switching back to drug-free aCSF (Figure 2A). In detail, we analyzed burst activity, taking into consideration three parameters (Figure 2B): (1) burst frequency; (2) number of events in burst; and (3) burst duration. Then, we examined whether any treatment-related changes in busting activity were coupled with intraburst single spike waveform (ISW) alterations. Since single spikes waveforms are characterized by a positive phase (hereafter named “peak”) and a negative phase (hereafter named “antipeak”), we took in consideration four fundamental parameters of ISW (Figure 2C): (1) peak amplitude; (2) duration of the positive phase (hereafter “time to peak”); (3) antipeak amplitude; and (4) duration of the negative phase (hereafter “time to antipeak”). The burst analysis was performed separately for both control and wash-out periods. For each recording it consisted of measuring the three parameters for each burst and then calculating the average for all the parameters. Afterwards, for all the burst parameters, the average values from each recording were also averaged for control and wash-out periods, respectively, for subsequent statistical analysis. Furthermore, ISW parameters were calculated from those bursts analyzed in the control and wash-out periods. ISW parameter values were averaged for each recording and as performed for burst analysis, combined into average absolute values from control and wash-out periods for statistical comparisons. The same procedure was adopted for burst and ISW analyses in the treatment period, considered as the last 5 min after switching to drug-included aCSF. All these parameters were considered as mean ± SEM and analyzed by a one-way ANOVA, comparing values from each treated period to the related control and wash-out periods. For all statistical tests used, the null hypothesis was rejected at a probability level (*P*) lower than 0.05.

## Results

### *In Vivo* MDA Model

#### Effect of Treatments on the Number of MDA Responses

**Figure 3:**
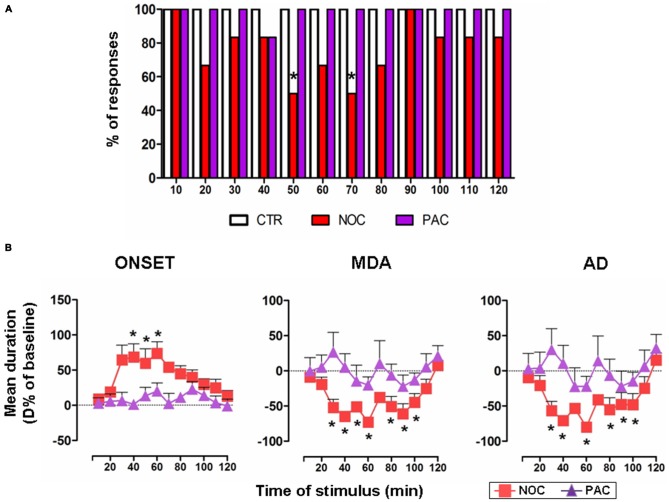
**(A)** The bar graph shows the percentage of responses to the AB stimulation recorded in the experimental groups, as indicated in the legend: vehicle-treated controls (CTR), nocodazole (NOC) and paclitaxel (PAC) during the progressive stimuli. **(B)** Effects of NOC (10 μM) and of PAC (10 μM) on the time course of MDA parameters during the progressive stimuli as in the legend. Each value represents the mean of D% ± SEM of each treatment per stimulus vs. baseline values. Within-treatment statistically significant D% is indicated for *P < 0.05 vs. baseline values.

A comprehensive bar graph showing the effects of each treatment on MDA responses is reported in Figure 3A.

In untreated and vehicle-injected controls repetitive AB stimulations always induced a MDA response along the experimental observation period, therefore in Figure 3A only the bar for vehicle-treated controls is reported.

NOC ICV injection reduced the % of responses to AB stimulation. In particular, NOC-treated group displayed a decrease in the percentage of responses to AB stimulation starting from 20th to 80th min, reaching a maximal effect at the 50th and 70th min when animals exhibited 50% of responses (*χ^2^* = 8.57, DF = 1, *P* = 0.0034).

Conversely, AB stimulations always induced a MDA response in animals treated with PAC.

#### Effect of Treatments on MDA Parameters

In untreated and vehicle-injected controls MDA parameters were not altered during the experimental session (Figure 1B).

In the group receiving NOC ICV injection, a within-treatment analysis revealed that the drug induced a significant increase in the time of onset (*F*_(12,60)_ = 2.29; *P* = 0.0209). Bonferroni post-test showed statistical significance from 40th to 60th min, with a maximum mean effect of +74.01 ± 40.19% at 60th min (from 4.26 ± 0.95 s to 7.10 ± 1.28 s; *P* = 0.005). Moreover, a reduction of MDA duration emerged (*F*_(12,60)_ = 7.068; *P* < 0.0001) vs. baseline, in particular from 30th to 100th min, with a maximum effect of −72.79 ± 15.11% at 60th min (from 23.24 ± 6.83 s to 5.41 ± 2.65 s; *P* < 0.0001). Similarly, the treatment induced a reduction in the AD duration (*F*_(12,60)_ = 5.469; *P* < 0.0001), namely from 30th to 100th min, with a maximum effect of −79.98 ± 13.34% at 60th min (from 17.57 ± 6.99 s to 2.67 ± 1.40 s; *P* < 0.0001). As for between-treatments comparisons of NOC vs. vehicle-treated group, a two-way ANOVA for the time of onset revealed significant main effects of stimulus time (*F*_(12,113)_ = 2.13, *P* = 0.0201) and treatment (*F*_(1,113)_ = 1.943, *P* < 0.0001), but not their interaction (*F*_(12,113)_ = 0.91, *P* = 0.53). The same analysis performed on MDA showed significant main effects of stimulus time (*F*_(12,113)_ = 6.291, *P* < 0.0001), treatment (*F*_(1,113)_ = 65.13, *P* < 0.0001) and their interaction (*F*_(12,113)_ = 4.277; *P* < 0.0001). Similarly, in the AD durations a significant main effect emerged for stimulus time (*F*_(12,113)_ = 4.508, *P* < 0.0001), treatment (*F*_(1,113)_ = 33.55, *P* < 0.0001) and their interaction (*F*_(12,113)_ = 3.208, *P* = 0.0006).

In contrast, a within-treatment analysis on PAC effect revealed no significant effects for any of the MDA parameters. A two-way ANOVA did not show a significant between-treatments main effect for stimulus time, treatment or their interaction for PAC-injected animals, when compared to vehicle group (Figure 3B).

#### Immunohistochemical Analysis after Nocodazole ICV Injection

**Figure 4:**
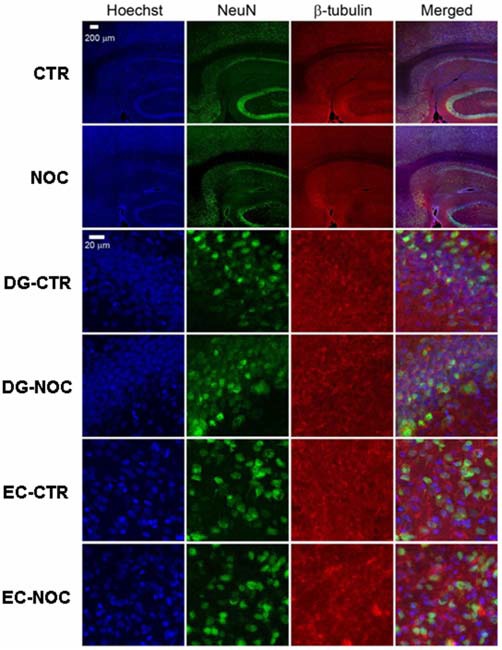
**The confocal projection images of coronal brain section showing immunohistochemical staining in vehicle-treated controls (CTR) and NOC-treated rats.** The hippocampal subregions analyzed are DG and entorhinal cortex (EC). Both NeuN (green) co-stained with β-tubulin protein (red), along with the nuclear stain Hoechst (blue) and the merged images are presented. Scale bar corresponds to 200 μm for 1st and 2nd row (10×), to 20 μm for the others (63×).

**Figure 5:**
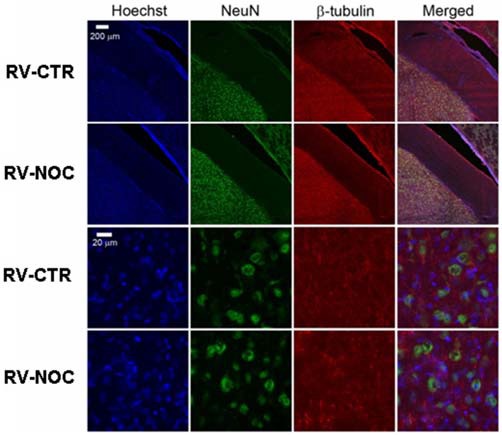
**The confocal projection images of coronal brain section showing immunohistochemical staining in vehicle-treated controls (CTR) and NOC-treated rats in the right cerebral ventricle (RV).** Both NeuN (green) co-stained with β-tubulin protein (red), along with the nuclear stain Hoechst (blue) and the merged images are presented. Scale bar corresponds to 200 μm for 1st and 2nd row (10×), to 20 μm for the others (63×).

**Figure 6:**
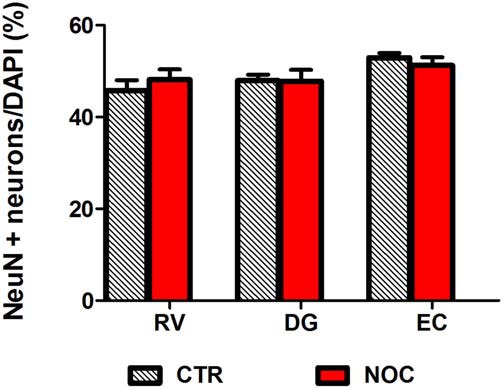
**Bar graph shows the ratio of NeuN positive neuron number/DAPI number in NOC-treated vs. controls (CTR) for each subregion: RV, DG and EC.** Values are expressed as percentage ratio ± SEM.

In the light of NOC effects, to evaluate the eventual neuronal loss due to excessive NOC-induced microtubule depolymerization, we conducted immunohistochemical staining of β-tubulin on coronal sections including DG, RV and EC. Figures 4, 5 report confocal projection images of cellular staining in control and NOC-treated brains. Both NeuN (green) co-stained with β-tubulin protein (red), along with the nuclear stain Hoechst (blue) and the merged images are presented. As shown in Figure 6, no significant changes in the NeuN/DAPI positive neurons ratio emerged from statistical analysis of treated vs. control animals in the RV (NOC: 48.14% ± 2.23; Control: 45.71% ± 2.32), DG (NOC: 47.77% ± 2.50; Control: 47.92% ± 1.32), and EC (NOC: 51.20% ± 1.84; Control: 52.92% ± 0.99), indicating that the NOC ICV injection has no neurotoxic effect in the detected brain regions.

### *In Vitro* HEBA Model

#### Induction of HEBA in Rat Brain Slices

**Figure 7:**
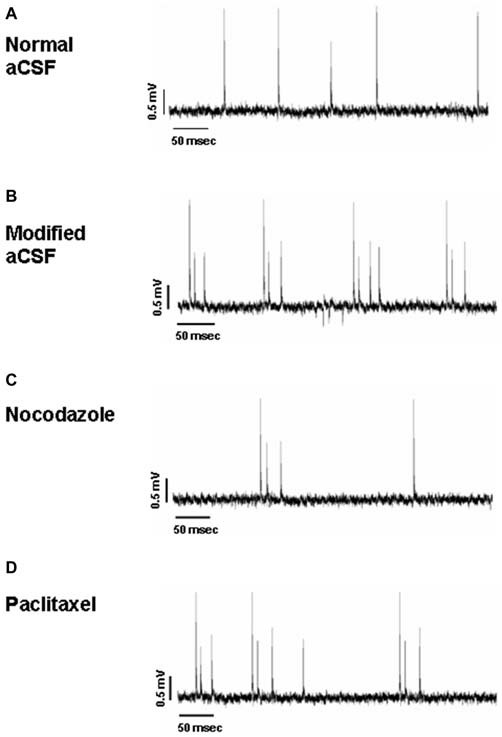
**Representative traces of hippocampal activity when slices are perfused with normal aCSF (A), modified aCSF (B), NOC (C) and PAC (D)**.

Hippocampal slices (*n* = 24) presented a stable, spontaneous single spike activity with normal aCSF (Figure 7A). The perfusion with the modified aCSF induced continuous burst activity in all slices (Figure 7B). Recordings were conducted on eight slices per treatment from hippocampal CA1 subregion. Each burst was characterized by clusters of extracellular potentials (1–4 mV amplitude). For all parameters taken into account, no statistically significant differences were observed between control values of burst activity in both NOC and PAC-treated slices.

#### Effect of Nocodazole at 10 μM and Paclitaxel at 10 μM on HEBA

**Figure 8:**
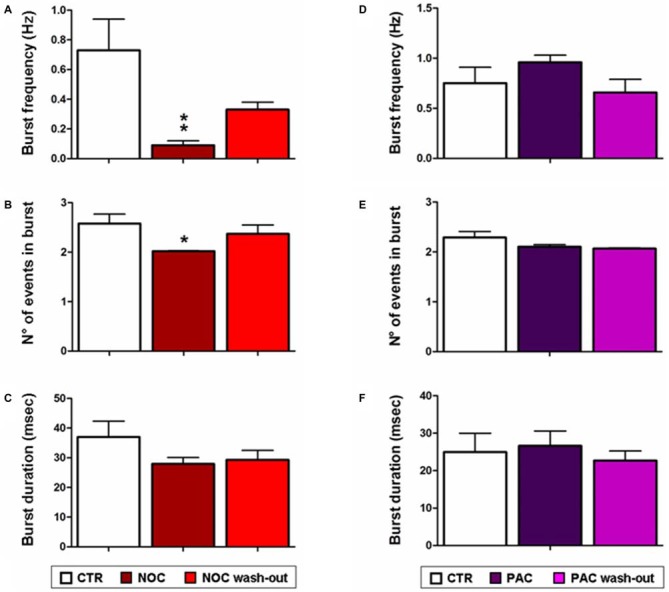
**Bar graphs showing NOC and PAC effects on burst parameters.** Each value represents mean ± SEM of, respectively, control (CTR), treated and wash-out periods, as indicated in the legend. Statistical significance is indicated for **P < 0.001 and for *P < 0.05 vs. control period. **(A,D)** Effects of NOC and PAC on burst frequency respectively. **(B,E)** Effects of NOC and PAC on the number (N°) of events in burst respectively. **(C,F)** Effects of NOC and PAC on burst duration respectively.

As for analyses on the effect of NOC on burst activity, a one-way ANOVA on burst frequency showed statistical significance (*F*_(2,23)_ = 6.60; *P* = 0.006), with a significant decrease during NOC 10 μM (from 0.73 ± 0.21 Hz to 0.09 ± 0.03 Hz; *P* < 0.001) vs. control. Furthermore, the number of events in bursts was significantly changed (*F*_(2,23)_ = 3.51; *P* = 0.048), particularly NOC reduced this parameter from 2.58 ± 0.19 to 2.019 ± 0.01 (*P* < 0.05) vs. control period. Lastly, burst duration was reduced following NOC treatment, though not-significantly (Figures 7C, 8). Noteworthy, after treatment with NOC in some recordings epileptiform activity was completely suppressed, though recovering during wash-out period. Figures 2A, 7 show a representative effect of NOC displayed both in compressed and extended time-scale traces.

Statistical analysis revealed that the parameters related to burst activity after PAC treatment were not significantly changed, though it seems that PAC slightly increases burst frequency and duration with respect to control period (Figures 7D, 8).

**Figure 9:**
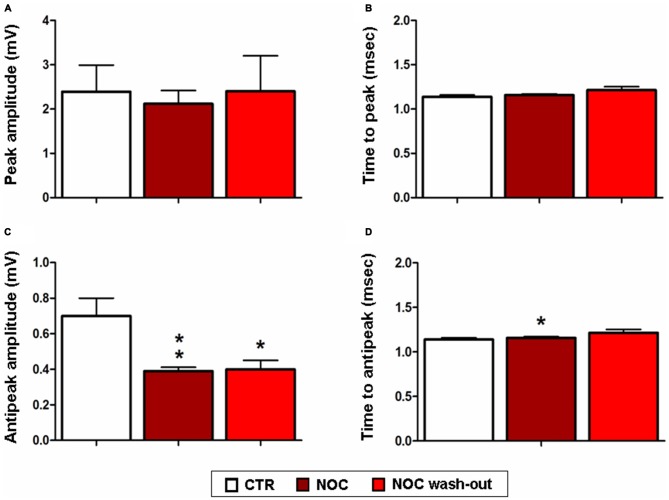
**Effects of NOC on ISW parameters.** Each value represents mean values ± SEM Statistical significances vs. control period (CTR) are indicated for **P < 0.001 and *P < 0.05. Bar graphs show the effect of treatments on the peak amplitude **(A)**, time to peak **(B)**, antipeak amplitude **(C)** and time to antipeak **(D)**.

Given that NOC resulted effective in modifying burst parameters, analyses on the ISW were performed on the related recordings. Statistical differences were found between control, NOC and wash-out period for the for the antipeak amplitude (*F*_(2,23)_ = 7.19; *P* = 0.0042) and for the time to antipeak (*F*_(2,23)_ = 4.67; *P* = 0.02). In detail, *post hoc* analysis showed that the antipeak amplitude significantly decreased during the administration of NOC 10 μM (from 0.7 ± 0.1 mV to 0.39 ± 0.021 mV; *P* < 0.01) and during the wash-out (from 0.7 ± 0.1 mV to 0.40 ± 0.005 mV; *P* < 0.05) respectively vs. control period. Furthermore, during NOC treatment the time to antipeak was significantly increased vs. control (from 1.49 ± 0.19 ms to 0.62 ± 0.026 ms; *P* < 0.05; Figure 9).

## Discussion

Dynamic changes in microtubule packaging system could represent the cutting edge of novel studies on the synaptic bases of epilepsy. Synaptic efficiency is strongly influenced by the microtubule cytoskeleton, exerting not only a mechanical action on cell morphology and plasticity, but also an operative role on transport, signaling and amplification of ion cloud (Brady et al., 2003; Craddock et al., 2010). For instance, cytoskeleton provides support to the trafficking of neurotrophic factors (Gardiner et al., 2011) and could be part of a system of conduction for the flux of charged ions along the axons (Craddock et al., 2010). The well-known “dynamic instability” of microtubules modulates synaptic activity and also neuronal response to insults (Poulain and Sobel, 2010). Consequently, a role played by microtubules in pathophysiological alterations of synaptic transmission, with a relapse on hyperexcitability phenomena, has been proposed (Furukawa and Mattson, 1995; Chuckowree and Vickers, 2003). To shed new light on this still elusive field, in the present research we tested drugs differently modulating microtubule polymerization, i.e., NOC and PAC, in two *in vivo* and *in vitro* models of drug-free epileptiform activity, the MDA and HEBA. This represents the main novelty of the current paper: the usage of drugs, widely administered as anti-mitotics (Jordan and Wilson, 2004; Gascoigne and Taylor, 2009; Endo et al., 2010), here applied to study epileptic states occurring in the hippocampus, a brain region where bioelectric activity can be modulated by agents of varied nature (Rizzo et al., 2009, 2014; Roseti et al., 2013; Allegra et al., 2015; Carletti et al., 2015).

NOC ICV injection protected animals in the MDA model from electrically-evoked paroxysmal discharge. In detail, this treatment decreased response to AB stimulation, starting from 20th min after drug administration. This suggests that NOC may intervene on activation threshold of epileptic discharge; moreover, it could mitigate the paroxysmal firing of DG neurons, based on the activity of glutamatergic reverberatory circuit of the hippocampal-parahippocampal system. In this perspective, NOC administration managed to modify MDA parameters, describing epileptiform severity. Indeed, in animals still responding to the stimulation, a marked reduction of the duration of both MDA and AD and a corresponding increase in the time of onset were observed. These data from MDA parameters hint that, once altered discharge is elicited in the DG, NOC ICV can attenuate its typical features. Whereas, the ICV administration of PAC did not influence MDA response nor discharge parameters. Then, immunofluorescence staining was performed in order to exclude that NOC efficacy in protecting from paroxysmal discharge was due to alterations that could harm cell integrity, eventually leading to a reduction in the number of neurons. Histochemical assay verified that NOC did not induce neuronal loss in the brain regions mainly involved in the MDA-related phenomena. This result seems to be consistent with the reported action of NOC as rather reversible and rapid with a relatively low toxicity (De Brabander et al., 1976; Lu et al., 2012). Nonetheless, a deeper histological analysis in future studies could further detail on putative morphological changes to neuronal population occurring after drug injection.

In order to support these findings with an *in vitro* approach, NOC and PAC were tested in the HEBA model. NOC was able to inhibit epileptiform burst activity, by diminishing burst frequency and the number of events in burst. Then, a further analysis on ISW data was carried out to better characterize drug-induced effects. We observed that alterations of hippocampal bursting activity were accompanied by a marked decrease in the amplitude and an increase in the duration of the negative phase. This is consistent with previous observations that lower spike amplitudes may result in a reduced probability to initiate bursts and also in a minor number of events within bursts (Harris et al., 2001). Accordingly, the increased duration of the negative phase could be responsible for the prolonged activity suppression. This is likely due to a longer hyperpolarization of neuron potentials, eventually reducing spike triggering. Importantly, burst activity seems to recover, though not always reaching control values, when NOC treatment is washed out. This also supports the verified absence of NOC neurotoxicity to the brain regions involved. In contrast, PAC did not seem to affect *in vitro* epileptiform activity. All considered, these data support the *in vivo* findings since NOC attenuates both genesis and severity of epileptiform activity.

The current results provide innovative evidence of the possible impact of microtubule cytoskeleton on hyperexcitability-based diseases. Noticeably, our experimental design exploiting two drug-free models of epileptiform activity (Stringer and Lothman, 1992; Sardo et al., 2012) allowed us to evaluate the results in an intact neuronal network, but also to exclude the influence of blood-brain barrier (BBB; Jefferys and Haas, 1982) and to appreciate whether any effect directly influenced hippocampal neurons. In this context, some molecular studies previously reported that, in kainate (KA)-induced epileptic state, altered microtubule formation contributed to aberrant neurogenesis in the DG with an increase in the gene expression of tubulin and microtubule-associated proteins (Represa et al., 1993; Pollard et al., 1994; Hendriksen et al., 2001; Sato and Abe, 2001). In contrast, drugs such as NAP, davunetide, that preserve microtubule functioning, protect against KA toxicity (Zemlyak et al., 2007, 2009). Therefore, manipulating the degree of microtubule polymerization could represent an intriguing strategy to act on hyperexcitability phenomena, though a comprehensive approach has not been adopted yet. The present research falls within this remit, by exploring the effects of microtubule-active agents in hippocampal epilepsy, and evidencing a clear antiepileptic effect only for NOC. In this regard, it has been reported that NOC and PAC differently regulate microtubule dynamics. On the one hand, NOC, depolymerizing microtubules, stimulates a morphological re-arrangement of neuritis (Chuckowree and Vickers, 2003). On the other hand, PAC is known to facilitate the polymerization of tubulin monomers, ultimately forming stable, non-functional microtubules (Gotaskie and Andreassi, 1994); this mechanism determined abnormalities of microtubule aggregation and growth within neurites in various animal models, producing PAC-induced aberrant structures (Letourneau and Ressler, 1984; Black, 1987; George et al., 1988; Theiss and Meller, 2000; Chuckowree and Vickers, 2003). These distinct outcomes could reflect different affinity of these drugs to the neuronal isoforms of αβ tubulin heterodimers, each responsible for specific processes such as cell viability, neurite outgrowth and protection against oxidative stress (Xu et al., 2002; Guo et al., 2010).

In addition, these alterations of microtubule dynamics may impact on other fundamental aspects of neuronal transmission including axonal transport. Actually, it was demonstrated that microtubule-based transport underpins the long-range transport into distal axons and dendrites (Puthanveettil et al., 2008). Disruption of intracellular transport is known to lead to axonal pathologies (Saxena and Caroni, 2007; Perlson et al., 2010; Millecamps and Julien, 2013). In this light, compounds affecting axonal transport machinery could modulate the neuronal hyperexcitability in mammalian brain, as also reported in previous studies on dimethyl sulfoxide, a strong microtubule stabilizer and fast axonal transport blocker (Donoso et al., 1977; Carletti et al., 2013). Moreover, it can be hypothesized that NOC may act on increased excitability occurring during paroxysmal events, by dissipating the excessive propagation of action potentials resulting from microtubule cytoskeleton ability to directly influence electrical currents and altered neurotransmission (Craddock et al., 2010). Intriguingly, this could be also likely explained by NOC modulation of fast mitochondrial transport which could reduce the amount of mitochondria in the synaptic terminals and ultimately impair substantial neurotransmission processes such as exocytosis, endocytosis and vesicle recycling (Morris and Hollenbeck, 1995; Miller et al., 2015).

In conclusion, our study show for the first time a clear effect of NOC in modulating acute electrophysiological paroxysmal activity, though further multidisciplinary data are surely required in order to better characterize a complete effectiveness profile. In this view, this microtubule-active agent would also deserve investigations to elucidate its implication in the hallmarks (e.g., axonal sprouting) of hippocampal neurogenesis occurring in chronic epilepsy (Chuckowree and Vickers, 2003; Wilson et al., 2012). Taken all this into consideration, focusing on new factors not strictly related to bioelectric features of synaptic transmission, microtubule cytoskeleton turns up amongst the possible mechanisms involved in hyperexcitability-based diseases.

## Author Contributions

FC, GG and X-AL conducted the experiments and data analyses. FC, VR, PS and GF designed the experiments. VR designed and directed the project. FC, PS, VR and GG wrote the manuscript with input from all the authors. All authors read and approved the final manuscript.

## Funding

This work was supported by grants of Italian Ministry of the University and the Scientific Research (M.I.U.R.), MIUR-UNIPA ORPA07BLYM, Rome, Italy.

## Conflict of Interest Statement

The authors declare that the research was conducted in the absence of any commercial or financial relationships that could be construed as a potential conflict of interest.
